# Developing a conceptual model for understanding caregiving experience and their impacts on quality of life for Chinese breast cancer family caregivers: A qualitative study

**DOI:** 10.1002/nop2.2139

**Published:** 2024-03-15

**Authors:** Chaoyue Gao, Min Li, Linfang Guo, Haoran Duan, Peili Zhang, Yongxia Ding

**Affiliations:** ^1^ School of nursing Shanxi Medical University Taiyuan China; ^2^ The Second Affiliated Hospital Zhejiang University School of Medicine Hangzhou China; ^3^ First Hospital of Shanxi Medical university Taiyuan China

**Keywords:** breast cancer, caregiving experience, family caregiver, quality of life

## Abstract

**Aim:**

The purpose of this study was to understand the caregiving experiences of breast cancer family caregivers and explore the profound impacts of those experiences on their quality of life.

**Design:**

A qualitative research method was used.

**Methods:**

We extended invitations to 23 family caregivers of outpatients and inpatients receiving breast surgery and oncology treatments in Taiyuan, China, to participate in semi‐structured interviews. The interviews were audio‐recorded and transcribed verbatim. Thematic analysis was employed to analyse the interview data.

**Results:**

Four themes and associated categories were identified: (1) changes in family dynamics, (2) the socio‐medical context, (3) interactions between family and society, (4) self‐efficacy and nine subthemes and their related categories, where virtually all participants expressed future uncertainty, emotional contagion, and personal challenges, and self‐efficacy had a moderating influence on the first three themes.

**Patient or Public Contribution:**

This study did not involve direct participation of patients or the public. However, their experiences and perspectives on caregiving were indirectly reflected through the insights provided by the family caregivers who participated in the interviews. Their valuable input contributed to a deeper understanding of the caregiving experience and its impact on the quality of life for Chinese breast cancer family caregivers.

## 1 INTRODUCTION

Breast cancer is the most common malignancy among women globally and remains the leading cause of cancer‐related deaths in this group (Arnold et al., 2022). Notably, China has experienced a significant and unique trend in breast cancer prevalence over the last 30 years, with an average annual growth exceeding 1.5%. Distinctively, Chinese patients tend to develop breast cancer at a younger age compared to global averages (Hiltrop et al., 2021). This specific trend underscores the importance of contextualizing breast cancer research within China, providing insights into how demographic and regional differences can inform and enhance global understanding of the disease.

The role of home nursing and family‐centred care is crucial in improving the quality of life for breast cancer patients, a concept widely recognized in both Chinese and international contexts (Leung et al., 2014; Mokhtari‐Hesari et al., 2018; Segrin et al., 2020; Townsend et al., 1990; Zhao et al., 2022). However, the increasing burden and psychological distress faced by family caregivers as a patient's condition worsens is a global concern, mirrored in China's experience (Bahrami & Farzi, 2014; Türkben Polat & Kiyak, 2023). The tendency to overlook caregiver burden in clinical practice is a worldwide issue, not limited to any single cultural or healthcare setting (Adelman et al., 2014; Giunta et al., 2022). This universal challenge highlights the critical need for research focused on family caregiver‐reported outcomes, aiming to provide a comprehensive understanding of caregiving's impact on quality of life, which is vital for developing effective early intervention strategies.

Data from several studies suggest that family caregivers of breast cancer may experience a poor influence on their mental health and quality of life as a result of the burden of care, which can be caused by psychological, occupational, financial, couple life and coping strategies (Gabriel et al., 2019). Psychologically, they may face emotional stress and anxiety from fluctuations in the patient's condition and uncertainty about treatment (Khanjari et al., 2014). Such psychological burden may adversely affect their emotional and mental health, thereby affecting the stability of daily life and interpersonal relationships. Additionally, occupational burden is a concern, as caregivers often need to find a balance between work and caring responsibilities, which may make them feel limited in their work and careers (Tao et al., 2022). At the same time, the financial burden on family caregivers may increase, especially if they are required to pay high medical bills or stop working to care for the patient (Kusi et al., 2020). Changes in couple life are also a potential source of burden, and caregivers may experience the evolution of couple relationships, emotional fluctuations and even feelings of social isolation, negatively affecting family and marital stability (Boamah Mensah et al., 2021; Noveiri et al., 2021; Thompson et al., 2022).

Although many qualitative studies have been conducted to assess the impact of caregivers' experiences, studies on the impact of breast cancer family caregivers' experiences within the Chinese cultural context remain limited. China's unique sociocultural environment, family structure and health perceptions may significantly impact caregivers' experiences and quality of life. This study addresses the limitations of previous research, which primarily focused on Western cultures, by offering a localized understanding. This understanding could lead to more specific, culturally sensitive recommendations for support and intervention.

This study employs a qualitative approach to deeply understand the caregiving experiences of Chinese breast cancer family caregivers within the cultural context of China. It aims to explore the profound impacts of these caregiving experiences on caregivers' quality of life. These insights are intended to inform researchers, healthcare institutions and practitioners, serving as a foundational reference for devising health education programs tailored to the needs of breast cancer family caregivers in China.

## 2 METHODS

### 2.1 Research design, participants and settings

From July to November 2021, participants were purposively sampled from breast Surgery and Oncology Outpatient Department of a tertiary hospital in Taiyuan, China. The caregivers who met the following criteria were invited: (1) age ≥18 years, (2) family caregivers of patients diagnosed with breast cancer and (3) providing unpaid care to patients. The exclusion criteria were as follows: family caregivers with communication impairment, hearing disorders. Data saturation determined study completion, with the study considered complete when no new themes could be identified from the dataset to add to our understanding of the phenomenon (Bowen, 2008). When the 21st participant finished her interview, no new information emerged. The first author (C.G.) and another graduate student (L.G.) approached the potential participants during clinical practice. After building rapport with interested participants, the first author explained the purpose and methods of this study and invited them to participate. All data are original data collected by this study and have not been published or used in other studies.

### 2.2 Data collection procedure

All interviews were conducted face‐to‐face by the author (REDACTED) and were arranged at a mutually convenient time and in a location of the respondent's choice, that is, the departmental office. The goal of the interview was to learn more about their caregiving experience and how it affected their life. If emotional shifts were felt throughout the interview, we would either stop the conversation or schedule the next interview depending on the participant's requests. During the whole procedure, just the interviewer and the participant were present, and the interviews were live recorded and transcribed with the participants' agreement. The topic guide (Table 1) was informed through literature review and research team discussion. The research team developed a first draft of the interview outline, selected three caregivers for pre‐interviews and adjusted the first draft based on the pre‐interview results to form the final interview guide.

**TABLE 1:** Exploring the caregiving experience of a family caregiver with breast cancer.

Opening question: ‘Please tell me about your experience of caring for a patient during diagnosis, treatment or recovery and how it has impacted your life’ Follow‐up questions How did you experience this role change in your family?
What did it mean to you in realizing that this was a long caregiving job? What is the biggest difficulty you are facing now? How did you solve it? What aspects of your life have been impacted as a result of your care?
Do you have any other sources of support? What aspects do you think these services have helped you? What other areas of stress are you facing besides caregiving? How did you feel when you realized that caregiving was affecting your life?

Data consisted of interviews and family caregiver diaries provided by participants. The opening questions were designed to encourage participants to speak freely and expose unexpected subjects and experiences, in order to foster immediacy (Dahlberg & Weiguang, 2008). Semi‐structured interviews were then conducted according to the interview outline. In addition to the interviews, the following data were obtained from the participants' records: age, relationship with the patient, occupation and education. The interviewees became the evaluators of the final results.

### 2.3 Data analysis

Interviews were analysed thematically. The interviews were transcribed into Chinese verbatim within 24 h of each interview. First, acquaintance with the text, processing empirical data and elaboration of the phenomenon's fundamental and particular meanings. Two researchers (Gao Chaoyue and Li Min) independently coded and divided units of meaning, which consisted of participants' statements, different words or phrases expressing the phenomenon, another senior researcher (Duan Haoran) reviewed all coding and quotations for completeness, accuracy and authenticity. Second, the study team discussed the meaning units, and the prototype themes were clustered to form subthemes and themes in more scientific and abstract language. In addition to the content, the research team focused on the interactions between the respondents and the interviewer. Respondents' verbal and emotional responses to questions and how the interviewer processed the interviewee's responses were included in the interview transcripts. In case of disagreements, the researcher reviewed the context verbatim, field notes and self‐reflection on the logic behind the codes or categories to reach a consensus. Third, the themes were defined to form conceptual models and explanations that reflect the core meaning and overall significance of the phenomena. We used consolidated criteria for reporting qualitative studies (COREQ) checklists for methodological quality assessment (Tong et al., 2007).

### 2.4 Ethical considerations

This study was conducted in accordance with the ethical guidelines in the Declaration of Helsinki (World Medical Association, 2013), and approval was obtained from the Ethics Committee of First Hospital of Shanxi Medical University (No:[2021] KY01). All participants were provided with detailed written and verbal information, clearly outlining the study's objectives, methodologies, any potential conflicts of interest, the voluntary nature of participation and the strict confidentiality of the data collected. Before joining the study, each participant signed an informed consent form and was reassured of their right to withdraw at any time. Strict confidentiality measures were implemented to protect the privacy of participant information.

## 3 RESULTS

### 3.1 Participant characteristic

Among the 25 individuals invited to participate, 23 expressed their willingness to be involved, while 2 caregivers declined the invitation. It is possible that caregivers facing heavier caregiving burdens were more aware of their caregiving‐related challenges, leading to their decision to opt‐out. Ultimately, a total of 23 caregivers actively participated in the interview process. Data saturation was achieved after conducting 21 interviews, ensuring comprehensive coverage of the research objectives. Of the 23 family caregivers enrolled (Table 2), 10 were the patients' husbands and 13 were the patients' offspring, with an average age of 39 years. Ten had a high school education or higher, including 2 with a bachelor's degree or higher.

**TABLE 2:** Basic information on the participant demographics.

Participants	Age (years)	Gender	Type of carer	Education	Race	Marital Status	Bereavement experience
P1	31	Female	Daughter	College or above	Han nationality	Married	Yes
P2	21	Male	Son	College or above	Han nationality	Married	No
P3	42	Male	Husband	College or above	National Minority	Married	Yes
P4	46	Female	Daughter	College or above	Han nationality	Married	Yes
P5	28	Female	Daughter	College or above	Han nationality	Married	Yes
P6	21	Male	Son	College or above	Han nationality	Unmarried	No
P7	32	Female	Daughter	High school	Han nationality	Married	No
P8	46	Female	Daughter	College or above	Han nationality	Married	Yes
P9	35	Female	Daughter	College or above	Han nationality	Married	Yes
P10	46	Male	Husband	Primary school and below	National minority	Married	Yes
P11	38	Male	Husband	Junior high school	Han nationality	Married	Yes
P12	44	Male	Son	High school	Han nationality	Married	Yes
P13	31	Male	Husband	Junior high school	Han nationality	Married	Yes
P14	43	Female	Sister	College or above	Han nationality	Married	Yes
P15	47	Male	Husband	Primary school and below	Han nationality	Married	Yes
P16	52	Male	Husband	High school	Han nationality	Married	Yes
P17	32	Male	Husband	Junior high school	Han nationality	Married	Yes
P18	33	Female	Daughter	College or above	Han nationality	Married	No
P19	45	Male	Husband	College or above	Han nationality	Married	Yes
P20	46	Male	Husband	College or above	Han nationality	Married	Yes
P21	27	Male	Husband	High school	Han nationality	Married	No
P22	48	Male	Husband	College or above	Han nationality	Married	Yes
P23	44	Male	Husband	High school	Han nationality	Married	Yes

### 3.2 Summary of themes

**FIGURE 1:**
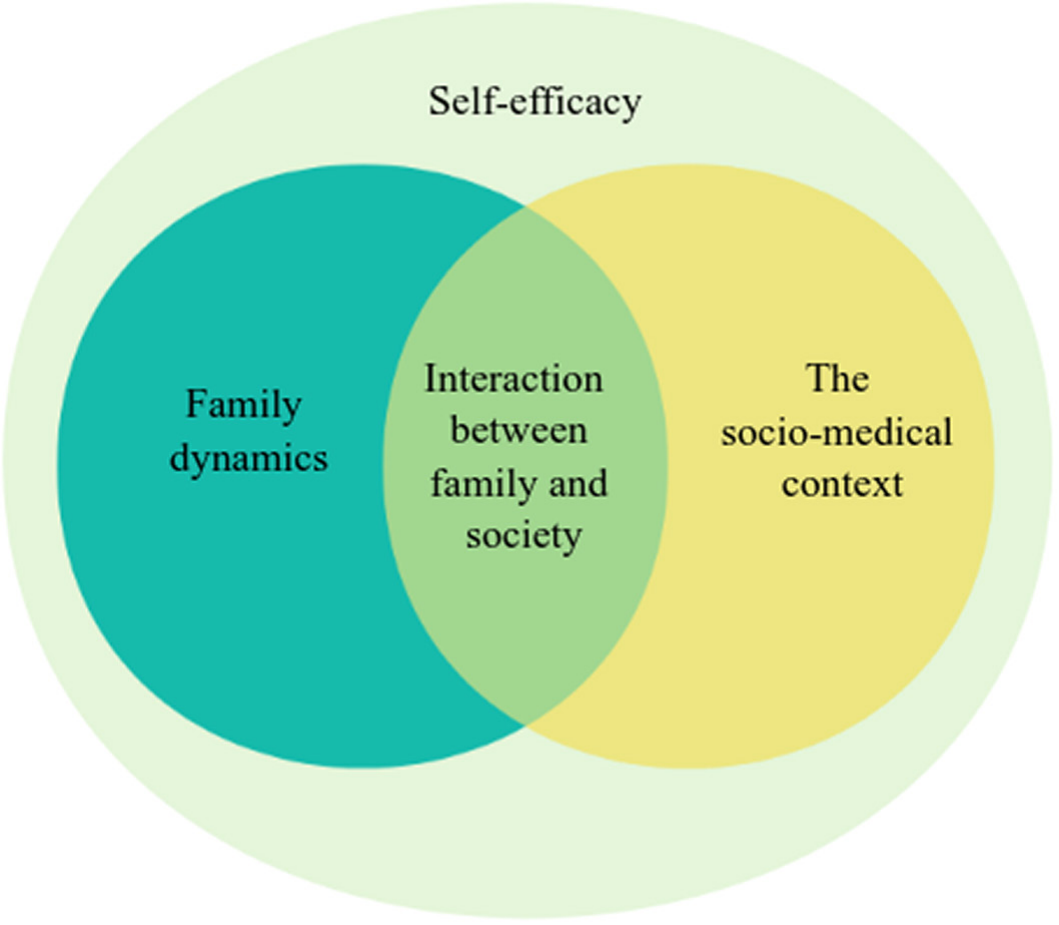
Schematic of quality of life themes valued by family caregivers.

**FIGURE 2:**
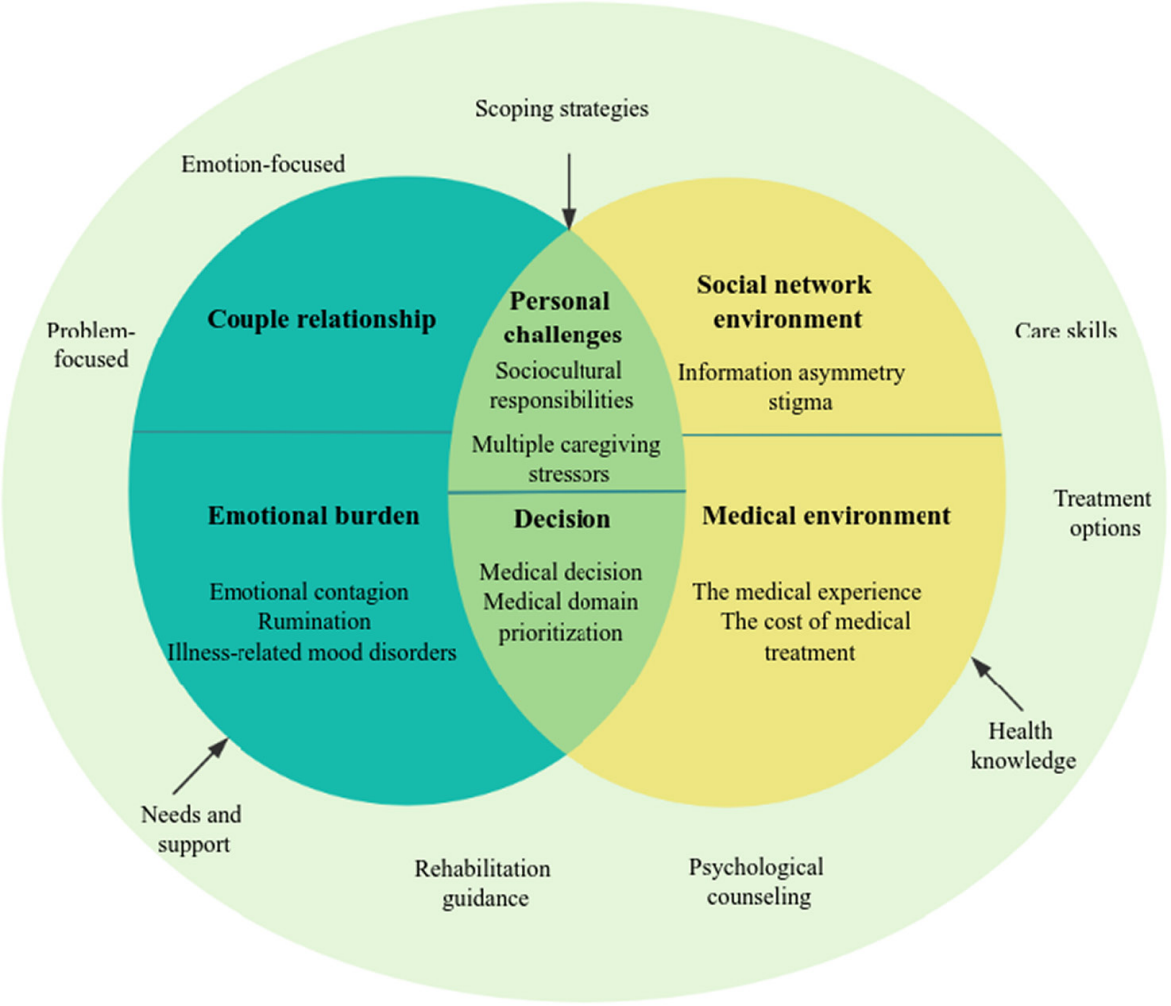
Schematic of quality of life subthemes and categories valued by family caregivers.

The experiences of family caregivers, providing insights into diverse aspects that affect their quality of life, were categorized into four main themes: (1) family dynamics, (2) the socio‐medical context, (3) interactions between family and society and (4) self‐efficacy (Figure 1). Family dynamics subthemes included family culture, couple relationship and emotional burden. The socio‐medical context subthemes included social network environment and medical environment. Interactions between family and society subthemes included personal challenges and decisions. Self‐efficacy subthemes included coping strategies, support and requirements and level of health knowledge. Additionally, beneath each subtheme, equivalent categories arose (Figure 2). Themes, subthemes and categories are found in Table 3. These themes, along with their sub‐themes, illuminated the specific challenges and resilience strategies employed by caregivers. From these insights, we developed a conceptual model to better understand how caregiving experiences impact the quality of life of Chinese breast cancer family caregivers. This model highlights the central role of self‐efficacy in mediating the effects of caregiving challenges on caregivers' quality of life. It illustrates a dynamic interplay between individual, familial and societal factors, emphasizing how caregivers' sense of self‐efficacy can buffer the stresses associated with their role.

**TABLE 3:** Themes, subthemes and example quotes from study participants.

Theme	Subtheme	Category	Example quote
Family dynamics	Couple relationship		‘I've learned the meaning of ‘mutual support’ through a series of exams… and I'm keeping track of our delight and happiness in life’. (P17) ‘…we often quarrel, saying that I do not care about her and our children, a good family that has been shattered by cancer, a chicken feather, full of devastation’ (P18)
	Emotional burden	Emotional contagion	‘Mother sick emotional irritability…, we take care of is also very difficult, unreasonable rage makes you want to go out regardless’. (P1) ‘I infected her with optimism, …, my family and I have gained growth’ (P7)
		Rumination	‘(The interviewee was in tears and speechless.) I regret that if I hadn't believed my wife's words and thought there was nothing wrong, I wouldn't have missed the treatment time…’ (P12) ‘I was extremely sad and devastated as I sat outside the surgery room waiting’. (P21)
		Illness‐related mood disorders	‘It was excruciatingly painful, and my mother paid little attention or refused to follow medical advice…’ (P11) ‘Two children are still minors, the wife has passed away, and the family will be dispersed… what should be done in the future?’ (P17) ‘…the probability of heredity exists in this genetic test daughter…double breakdown’ (P18)
The socio‐medical context	Social network environment	Information asymmetry	‘Search the Internet for this information… terrified… the more you know, the more scared you will be’. (P12) ‘Mom believes the patient group that there are not so many tests and that the doctor is only interested in making money’. (P4)
		Stigma	‘Family caregiver of breast cancer patients are my permanent label…boyfriend broke up with me because of it’ (P12)
	Medical environment	The medical experience	‘…feeling the care and encouragement of the doctors…we can better go to today’ (P7) ‘After waiting all night for a chemo plan, the doctor delivered it in 2 min and didn't have time to explain it’. (P7)
		The cost of medical treatment	‘…every time I awoke at 5 a.m., took a taxi to the hospital at 6 a.m., registered, read reports, booked a bed, pre‐chemotherapy tests, chemotherapy, and then was discharged in the afternoon, which exhausted me’. (P6) ‘Despite being accepted to the hospital, beds were not available until the day before surgery, so we had to locate a hotel until then’. (P4) ‘The cost of treatment was cobbled together…the agony of not being able to cry as we looked for medical help and sought funds was agonizing’. (P16)
Interaction between family and society	Personal challenges	Sociocultural responsibilities	‘A “filial son” is tolerant in a way that others are not’. (P1) ‘…My two brothers were frightened and fearful as a result of our parents' education and management style, and they didn't know how to care for my mother… I basically took on all of my mother's tasks in terms of caring’.(P5) ‘…They always seemed to wear my mother down, and I felt awful for her…’ (P5) ‘My wife needs to be with me throughout her illness, I have a primary school‐aged boy at home, and I can't miss work… When the family is aware of the problem, they refuse to assist and frequently complain’. (P16)
		Multiple caregiving stressors	‘My wife was in the hospital preparing to have open heart surgery, and my father was in Wuxi about to have heart bypass surgery that night, so I couldn't hold out much longer’. (P15)
	Decision	Medical decision	‘You're under a lot of psychological strain; you have to make decisions for each treatment and can't afford to skip any, and the tension and depression aren't something you want to remember’. (P7)
		Medical domain prioritization	‘…I couldn't move my back while waiting for the operation… I rushed back after receiving a brief shot’. (P4)
Self‐efficacy	Scoping strategies	Emotion‐focused	‘Sometimes it is faith that conquers all’ (P16)
		Problem‐focused	‘I looked up the NCCN treatment guidelines, which was a lot less of a detour…’ (P7) ‘…Running, meditation, reading’ (P7)
	Health knowledge	Care skills	‘If she has a poor response and refuses to eat, you can't just give it to her…prepare the porridge ahead of time…feed her one bite at a time…drink plenty of water to help the drug metabolize through urine…’ (P6) ‘…fortunately, I work at a hospital, so I can assist my mother with the medicine changes at home’. (P6)
		Treatment options	‘I would advise you to visit a few more hospitals and see a few more doctors to gain a better understanding of the various illnesses’. (P12)
	Needs and support	Psychological counselling	‘Professional psychological counselling is required to ensure that the mother does not refuse treatment in the future due to negative reactions’. (P11)
		Rehabilitation guidance	‘Our have no idea how to recover from surgery or supplement nutrition, and my mother doesn't pay attention’ (P9)

### 3.3 Theme 1: family dynamics

Although each participant's family education, personality, cohesiveness, and resilience are unique, virtually all participants perceived some degree of disruption in their family homeostasis, associated with the diagnosis, therapy, disease progression and even recurrence of breast cancer. In the narratives of the family caregivers, we observed recurring themes indicating that factors commonly associated with family stability were intricately linked with their perceived quality of life:

#### 3.3.1 Sub‐theme 1: couple relationship

The experiences of illness on the couple's relationship has both positive and negative aspects. It is a mutually reinforcing dynamic, where a good relationship and supportive behaviour of the spouse help to reduce the psychological stress of both partners, and where the ordeal of the disease makes the husband appreciate the patient more and take the initiative to ‘record the joys and pleasures of life’. Conversely, especially for young couples, changes in sex life, personal values and physical appearance of the patient make caregivers feel ‘powerless’ and describe their lives as ‘broken and devastated’.

I felt compelled to document the visit and express my initial disbelief regarding the malignancy of my wife's condition. Little did I know that this act of recording would prove invaluable after her eventual diagnosis. Just as Mayo's book suggests, the recorded account served as a valuable tool for tracking our emotions and preserving memories. Amid our already challenging phase in life, it has granted us a deeper understanding of life's priorities and the people who matter most to us. Moreover, it has provided us with spiritual insights and other profound aspects that some individuals may only discover in the twilight years of their lives. Tomorrow, I am scheduled to have my wound dressing changed. Wishing you all the best! (p22).

#### 3.3.2 Sub‐theme 2: emotional burden

Although the emotional burdens of the individuals were diverse, the majority of them stemmed from three areas: emotional contagion, rumination and illness‐related mood disorders. Family caregivers perceived mutually supportive emotional interactions as a way for both partners to ‘grow with each other’, whereas the patient's ‘sensitivity, suspicion and irritability’ placed an emotional burden on the caregiver and led to family tension. Family caregivers may regurgitate their ideas into a troubled state of mind when they create links between past occurrences and the sickness, and the deeper the relationship, the more intense the distress. We also discovered that family caregivers suffer from the same disease‐related emotional disorders as patients, such as helplessness after diagnosis, worry about adverse treatment reactions, feeling of powerlessness about poor treatment compliance and fear caused by various uncertainties such as disease genetic inheritance, while young caregivers' life states are more affected by uncertainty and often feel ‘double collapse’. That is the dual stress of managing one's own emotions while simultaneously attending to the needs of the patient. In such a state, young caregivers may experience exhaustion and find it difficult to effectively navigate their own emotions and the challenges associated with the illness.

These days, I find myself repeatedly pondering the same question: Why? What did we do wrong? My wife has always enjoyed excellent health, rarely falling ill even with minor colds throughout the year. It is only since her cancer diagnosis that our lives have been turned upside down. The slightest shift in her health condition fills me with overwhelming anxiety. I am aware that dwelling in such a state is not beneficial, but breaking free from this cycle feels like an insurmountable challenge. (p19).

### 3.4 Theme 2: the socio‐medical context

#### 3.4.1 Sub‐theme 1: social network environment

In our study, caregivers' experiences within the social network environment were deeply influenced by factors like ‘information asymmetry’ and ‘stigma’. Almost all caregivers will turn to a network platform, such as Baidu, paste bar or a breast cancer patients mutual help group, to resolve disease‐related concerns. These platforms can provide all kinds of information for patients and caregivers, but they are also affected by self‐judgement ability and disease specificity, resulting in a situation similar to that of ‘Baidu medical treatment’, resulting in a crisis of confidence between doctors and care difficulties. Illness stereotypes can ‘label’ carers and significantly impact the marital status of young female caregivers.

I recall conducting online research regarding this topic, and the information I found indicated a poor prognosis for this particular cancer stage. It truly frightened me, and as I delved deeper into my research, my fear grew. It took me a considerable amount of time to gradually come to terms with the situation. We chose not to disclose too much information to my mother as it would have been cruel for her to confront the survival and recurrence rates. Fortunately, she did not become overly fixated on these matters. (p12).

#### 3.4.2 Sub‐theme 2: medical environment

In the narratives of our participants, the socio‐medical context appeared to influence their quality of life in two primary areas: the medical experience and the cost of medical treatment. It is difficult to register in China due to societal issues such as a lack of quality medical resources and the inversion of medical services, and there is ‘I waited in a long queue, but the doctor only spoke to the patient for 2 minutes’. Medical attention and encouragement, according to the participants, might provide ‘greater hope in life’.

Medical costs include money and physical costs. At present, in order to improve the utilization rate of beds, hospitals in our country carry out pre‐hospitalization and daytime chemotherapy policies, and frequent access to hospitals causes ‘fatigue ‘of caregivers. In addition, transportation and accommodation expenses, including medical expenses, are also required.

Upon our arrival at the inpatient unit, we encountered a lack of available beds, and we had to wait until nearly 3:30 pm for beds to become available and for us to proceed with the check‐in process. Another issue arose during the hospitalization due to my mother's foreign health insurance status. Having interacted with many patients' families over the years, I used to empathize with their feelings, but now that I'm personally experiencing it, I realize how challenging it truly is. I feel the urge to explode and express my anger, but I understand that venting these emotions won't solve the problem. I engaged in a conversation with the staff at the window for over an hour, and eventually, the necessary procedures were completed. (p4).

### 3.5 Theme 3: interactions between family and society

#### 3.5.1 Sub‐theme 1: personal challenges

Family and social interactions can present caregivers with many challenges, commonly found in younger caregiving populations. These challenges include sociocultural responsibilities and multiple caregiving stressors.

The traditional Chinese filial piety culture imposes the label of ‘respect for relatives, foster care, serve illness, and be excellent at death’ on both the external environment and the carers themselves. The label of ‘filial son’ restricts participants' conduct while also promoting the establishment of familial inequality. The participants select ‘tolerance’ to make it an unconditional givers, especially when the patient is ‘unreasonably troublesome’. Almost everyone in the study believes that role disorder causes them to feel ‘tired’ and ‘physically uncomfortable’.

Female participants reflected on their caregiving role during the caregiving period and believed that they had unconsciously assumed more family responsibilities as a result of their family culture and education, which also gave them a tendency to take care of the patient's family, children and relatives in particular. Furthermore, in households where the conventional belief is ‘men manage external affairs, women internal’, female breast cancer sufferers are responsible for practically all aspects of family life, including caring for their children, serving their spouses and honouring their in‐laws. As a result, hatred and unfairness have arisen as a result of ‘feeling unworthy for their mother’ and a lack of brother support. This familial milieu, the carers feel, ‘makes them tired’.

Multiple caregiving stress was an unexpected category, and we found that five participants whose families had multiple incidental family events during the same time period of time and become caregivers of multiple patients described their lives as ‘ overwhelmed’ and ‘broken’.

During my mother's hospitalization, I, along with my husband, took on the role of caregivers. However, her illness had a negative impact on her temperament, leading to frequent suspicions about the authenticity of the medicines and supplements I purchased for her. As a result, our relationship became strained and tension arose between us. (P9).

#### 3.5.2 Sub‐theme 2: decisions

This is a negative theme that varies depending on the level of knowledge of the illness, literacy, personality, physical condition, previous family status and professional background of both parties. It includes medical decisions, life domain conflict and medical domain prioritization.

Most participants believed that making their own medical decisions reduced the psychological stress and physical strain of going to the doctor, but they felt ‘stressed and depressed’ and even in a moral dilemma. In particular, caregivers who opted to conceal their loved one's condition were significantly influenced by their living circumstances. They expressed, ‘This decision cannot be wrong, as all the psychological burden falls solely on me’. This tendency was particularly prominent among young male caregivers who frequently made sacrifices, forsaking their employment, leisure time and even personal growth. They described their role in caregiving as akin to that of ‘mercenaries’.

Due to the deteriorating condition of my wife and my demanding work schedule, I found it increasingly challenging to provide adequate care for her and our children. As a result, she had to relocate and live with her mother for better support. Additionally, the exorbitant medical expenses forced me to frequently change jobs, without a stable career plan, akin to a mercenary, constantly moving around the country in search of better financial opportunities. (P23).

### 3.6 Theme 4: self‐efficacy

The interviews found that self‐efficacy was related to the way participants selected coping strategies, their level of health knowledge and the degree to which their self needs and support were met.

Caregivers use a variety of coping strategies and behaviours when dealing with family, social and self‐stress. Participants' coping strategies were classified as emotion‐focused or problem‐focused, according to Lazarus' stress adaptation theory, but most participants stated that the coping strategies they used could only have short‐term effects, such as using ‘prayer’ to gain temporary psychological satisfaction. Higher‐knowledge participants were able to provide better personal guidance and care experiences, and they were able to save money on medical bills to some extent. Almost all participants expressed a wish to ‘return to their previous lifestyles’ and ineffectiveness in coping with present life inequalities and a need for psychological support, illness‐related information and rehabilitation advice.

Some individuals in the support group shared stories of praying for their wives with similar intentions, which inspired me to pray for my beloved as well. I fervently wished for her to have a long and fulfilling life, envisioning a future where we would grow old together and find happiness. (p20).

I cannot pinpoint when the doctor‐patient relationship became so strained, leaving behind no trace of trust. However, I would like to emphasize the importance of placing your faith in the medical professionals rather than relying on online sources like Baidu or following anecdotal advice from other patients. Despite my frustration, I took solace in the authoritative ‘Breast Cancer Guide’ that I obtained promptly. Equipped with this valuable resource, I encouraged my mother to consult it herself and assess whether any unnecessary tests were being recommended by her doctor.

(p4).

## 4 DISCUSSION

This qualitative study adopted a one‐on‐one in‐depth interview among 21 patients. Four themes related to quality of life emerged from the study: changes in family dynamics, socio‐medical environment, family‐society interactions and self‐efficacy. Significantly, the finding indicated that self‐efficacy effectively moderates the aforementioned themes, contributing to the preservation of a dynamic equilibrium between caregivers and their lives. A conceptual model was developed in which emotional contagion, sociocultural responsibilities, multiple caregiving stressors and personal challenges were mentioned by almost all participants. We need to reflect on the meaning behind these phenomena, the context and the ways in which they emerge. This reflection will help make healthcare more focus on the lives of family caregivers. Additionally, it can guide family care and improve the effectiveness of quality‐of‐life interventions.

The findings revealed that emotional contagion significantly influences the emotional experiences of family caregivers. Additionally, it was observed that patients' emotional states and behavioural attitudes impact caregivers' quality of life. This aligns with the interdependence model of suffering experienced by cancer patients and caregivers, as outlined in the research by (Segrin et al., 2020). Furthermore, these findings corroborate the theory of emotional contagion process (Hatfield et al., 1993). However, we discovered that family culture is a moderating factor in the emotional contagion process. In the Chinese context, filial and gender cultures tend to contribute to inequity in family relationships. This inequity, in turn, has a detrimental impact on caregivers' emotional experiences, as shown in the conceptual model we developed. Wróbel and Imbir (2019) argued that emotional contagion is not blind. They explored the influence of social factors on emotional contagion and its mechanisms within the framework of dual process theory. According to this theory, the receiver reflects on the sender's emotions and is subject to automatic emotion correction influenced by cognitive resources, motivation and attention (Eslinger et al., 2021; van Kleef & Côté, 2022). We may do more data mining on emotional contagion in the future. This exploration aims to discover how we can improve the cognitive abilities of family caregivers. By doing so, we intend to initiate a reflective model that encourages them to use a positive behavioural motivation to save themselves from negative emotions and improve both parties' quality of life.

Male caregivers face distinct challenges influenced by gender identity and notions of masculinity. They often assume the obligation to actively care for and support female patients, including providing emotional support. This sense of duty frequently leads them to alter their lifestyle and career pursuits. Consequently, they tend to neglect their own care and support needs (Rosdiana & Afiyanti, 2022). Nelson C. Y. Yeung et al (Yeung et al. 2018) argued that male spousal caregivers, influenced by Chinese cultural norms, may experience caregiving guilt. This guilt is associated with marital satisfaction and the endorsement of traditional gender roles. However, the scope of this study was small, and it yielded no significant findings in this area. Therefore, future research is needed for an in‐depth exploration of male caregivers.

Multiple caregiving stressors were an unexpected finding in the study. We referred to cancer diagnosis as a family contingency. Caregivers facing multiple family contingencies, either simultaneously or sequentially, experienced ineffective coping and empathic fatigue. They also reported physical discomfort, including loss of appetite and decreased sleep quality. These findings consistent with other studies finding physical aspects of caregivers' quality of life (Chang et al., 2007; Stenberg et al., 2010).

According to the conceptual model, self‐efficacy moderates the burden of caregiving due to changes in family dynamics, the socio‐medical context and interaction between family and society. Caregivers with high self‐efficacy are able to effectively cope with stress and improve their quality of life. The study found that coping strategies, health literacy and needs are the three main aspects that influence self‐efficacy. Additionally, modulating quality of life, caregiving burden and disease management skills have greater research potential as important components of psychosocial interventions (Arenella & Steffen, 2020; Jacobs et al., 2020; Kizza & Maritz, 2019). Samia et al. (2019) designed the savvy advanced psychoeducation program that found significant improvements in family caregivers' self‐efficacy and depressive symptoms. Caregivers can improve their self‐efficacy through conscious interventions that are well suited for inclusion in our supportive care system (Levesque et al., 2022; Yeung et al., 2020). Currently, the self‐efficacy status of family caregivers with breast cancer in China is not receiving sufficient attention, and the development of specific intervention programs is an important topic for us to accomplish in the future.

This qualitative study, through its exploration of quality of life among Chinese breast cancer family caregivers, develops a conceptual model that underscores the crucial interplay between social support, family dynamics and individual self‐support, revealing significant insights into the unique caregiving experiences within the specific sociocultural and healthcare context of China. From an international nursing perspective, it not only highlights the challenges and dynamics unique to the Chinese setting but also contributes to the global discourse on family caregiving. By demonstrating the flaws within China's healthcare support system and emphasizing the necessity for culturally sensitive care practices, this study advocates for supportive interventions that are thoughtfully tailored to the nuanced needs of family caregivers across diverse cultural landscapes. The findings and the developed conceptual model enrich our global understanding of family caregiving, underscoring the importance of a supportive ecosystem that accommodates the complex interplay of social, familial and personal factors in caregiving experiences.

## 5 CONCLUSION

This study aimed to examine the impact of caregiving on the lives of family caregivers, identifying key issues that arise from this experience. The findings revealed four prominent themes related to quality of life: changes in family dynamics, socio‐medical environment, family–society interactions and self‐efficacy. Notably, self‐efficacy effectively moderates the aforementioned themes, ensuring a dynamic equilibrium between caregivers and their own lives. Subsequently, a conceptual model was developed to illustrate the caregiving experience and its influence on quality of life. Based on these findings, it is imperative to underscore the significance of self‐efficacy in enhancing the quality of life, urging the integration of self‐efficacy into supportive care interventions to foster innovation and effectiveness.

## 6 LIMITATION

Reflecting on this study's limitations, it is notable that its setting, confined to a single hospital in Taiyuan, China, may not fully represent the diverse caregiving contexts across the country. The methodology, relying on semi‐structured interviews, introduced a specificity that, while rich in detail, limits the findings' applicability across different caregiving scenarios, including the unique experiences of parents as caregivers—a group not included due to their distinct sociocultural roles in China. Further, we failed to distinguish between family caregivers at each stage of illness. Ethically, while rigorous measures were taken to ensure participant confidentiality and informed consent, the sensitive nature of the subject matter might have influenced the depth of disclosures. These aspects suggest avenues for future research to address these gaps and enhance our understanding of caregiving in diverse settings.

## AUTHOR CONTRIBUTIONS

Conceptualization: Chaoyue Gao, Min Li, Linfang Guo, and Yongxia Ding; Formal analysis: Chaoyue Gao and Peili; Methodology: Haoran Duan, Min Li, Linfang Guo, and Yongxia Ding; writing—original draft: Chaoyue Gao; writing—review and editing: Min Li and Haoran Duan.

## FUNDING INFORMATION

The authors declare that no funds, grants or other support were received during the preparation of this manuscript.

## CONFLICT OF INTEREST STATEMENT

The authors declare that they have no competing interest.

## ETHICS STATEMENT

This study was conducted in accordance with the ethical guidelines in the Declaration of Helsinki, and approval was obtained from the Ethics Committee of First Hospital of Shanxi Medical University (No:[2021] KY01).

## INFORMED CONSENT

Informed consent was obtained from all individual participants included in the study.

## OPEN ACCESS

This article is distributed under the terms of the Creative Commons Attribution 4.0 International License (REDACTED), which permits unrestricted use, distribution and reproduction in any medium, provided you give appropriate credit to the original author(s) and the source, provide a link to the Creative Commons license, and indicate if changes were made.

## Data Availability

The data that support the findings of this study are available from the corresponding author upon reasonable request.
